# 

**Published:** 1893-09-23

**Authors:** 


### CONTENTS.

**The British Association** - - - - - - - - 401

**Around the Hospitals** - - - - - - - - - - 402

**The Hygiene and Advantage of Illness** - - - - 403

**Contemporary Theory and Research** - - - - - 404

**Famous Poisoners in Fiction.**—V.—The Poison Maid - - 405

**The Hospital Clinic**—

HUDDERSFIELD INFIRMARY.—Fracture and Dislocation of the Spine. By Norman Porritt, M.R.C.S., &c. - - - - 409

INFANTILE PARALYSIS. By B. Rogers, B.A., M.D., &c. - - 411

THE ROYAL SOUTHERN HOSPITAL, LIVERPOOL. Treatment of Agne - - - - - - - - - - - 411

INUNCTION IN SCARLET FEVER - - - - - - - - 412

**Gleanings in Science and Medicine** - - - - - 406

**Annotations.**—The New Coachman of the British Association—Is Vivisection Moral ?—More Wicked than Sacrilege - - - 407

**Notes and News** - - - - - - - - - - - - 408

**The Institutional Workshop**—

HOSPITAL ADMINISTRATION.—IV.—Special Hospitals : Lying-in and Women - - - - - - - - - - - 413

THE STORY OF THE INSANE FROM YEAR TO YEAR - - - - 414

THE DEATH AT THE LONDON HOSPITAL - - - - - - 415

PRACTICAL DEPARTMENTS.—Boot Warmer and Drier - - - 415

CONSTRUCTION NOTES - - - - - - - - - - 416

**Scraps and Gleanings** - - - - - - - - - 416

**The Book World of Medicine and Science** - - - - vii

**Medical Vacancies and Appointments** - - - - - ix

### EXTRA SUPPLEMENT.—THE NURSING MIRROR.

**News from the Nursing World.**—Our Examinations—Teachers and Taught—Elastic Limits—Critical Observers—Well Cared For—Worthy of Imitation—A French Doctor's Waiting-room—India—Australia—Our American Sisters— ccli

**Trained Nursing in Berlin**—The Victoria House - - ccliii

**Wants and Workers** - - - - - - - - ccliii

**Nursing in Paris.** By a WANDERING NURSE.—First Impression of the Hotel Dieu - - - - - - - - cclv

**District Nursing.**—By an ASSOCIATION SUPERINTENDENT. cclvi

**The Parish Nurse.** II.—Her Position and Duties - - ccliv

**Nursing in Ireland.**—County and City of Cork Hospital for Women and Children - - - - - - - - - - cclv

**Everybody's Opinion.**—Is Vivisection Justifiable—"Nurses and Dress"—Nursing Uniforms—Pedestrians and Cyclists cclvi

**The Muses' Looking Glass.**—Annual Holidays—"Times of Refreshing" - - - - - - - - - - - - cclviii

**Notes and Queries** - - - - - - - - - - - cclviii

**The Book World for Women and Nurses** - - - - cclix

